# CRISPR/Cas: From Tumor Gene Editing to T Cell-Based Immunotherapy of Cancer

**DOI:** 10.3389/fimmu.2020.02062

**Published:** 2020-09-29

**Authors:** Mohammadreza Azangou-Khyavy, Mobina Ghasemi, Javad Khanali, Melika Boroomand-Saboor, Monire Jamalkhah, Masoud Soleimani, Jafar Kiani

**Affiliations:** ^1^Student Research Committee, Shahid Beheshti University of Medical Sciences, Tehran, Iran; ^2^Department of Biotechnology, College of Science, University of Tehran, Tehran, Iran; ^3^Hematology Department, Faculty of Medical Sciences, Tarbiat Modares University, Tehran, Iran; ^4^Oncopathology Research Center, Iran University of Medical Sciences, Tehran, Iran; ^5^Department of Molecular Medicine, Faculty of Advanced Technologies in Medicine, Iran University of Medical Sciences, Tehran, Iran

**Keywords:** CRISPR, cancer treatment, gene therapy, cancer immunotherapy, CAR T cell therapy, genome-wide screening assays, oncolytic virotherapy

## Abstract

The clustered regularly interspaced short palindromic repeats system has demonstrated considerable advantages over other nuclease-based genome editing tools due to its high accuracy, efficiency, and strong specificity. Given that cancer is caused by an excessive accumulation of mutations that lead to the activation of oncogenes and inactivation of tumor suppressor genes, the CRISPR/Cas9 system is a therapy of choice for tumor genome editing and treatment. In defining its superior use, we have reviewed the novel applications of the CRISPR genome editing tool in discovering, sorting, and prioritizing targets for subsequent interventions, and passing different hurdles of cancer treatment such as epigenetic alterations and drug resistance. Moreover, we have reviewed the breakthroughs precipitated by the CRISPR system in the field of cancer immunotherapy, such as identification of immune system-tumor interplay, production of universal Chimeric Antigen Receptor T cells, inhibition of immune checkpoint inhibitors, and Oncolytic Virotherapy. The existing challenges and limitations, as well as the prospects of CRISPR based systems, are also discussed.

## Introduction

**FIGURE 1:**
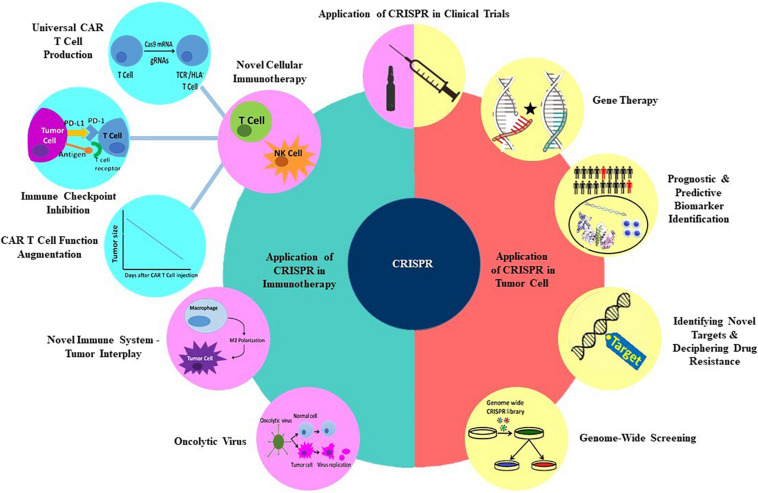
Applications of CRISPR technology in multiple aspects of cancer treatment.

In recent years, various genetic manipulation techniques have been developed which involve DNA repair mechanisms that incorporate site-specific modifications into a cell’s genome. These techniques have made diverse genome alterations in a site-specific manner possible, as they are able to edit tumor cells’ genome to induce apoptosis, reduce drug resistance, and restore mutant genes. They also can be employed in immune system genetic manipulation to potentiate antitumor immune responses (1–4). Clustered Regulatory Interspaced Short Palindromic Repeats (CRISPR) is a novel mammalian cells’ genome editing technique derived from archaeal and bacterial antiviral defense systems. Due to its exceptional potential and efficacy, this platform has defined a new era, tipping the balance of cancer treatment in favor of genetic manipulation of the tumor and immune cells. CRISPR is preferentially exploited to interrogate various genes and signaling pathways’ functions, leading to the discovery of new therapeutic targets. In this review, we will discuss the CRISPR technique and explore its recent applications in discovering new targets in cancer therapy and overcoming different hurdles of cancer treatment, such as oncogenes, epigenetic dysregulations, and drug resistance. We will also dissect its redemptive potential in immunotherapy. The subtitles that will be discussed later in this review are shown in Figure 1.

## Genome Editing Techniques: ZFNs, Talens, and CRISPR

**FIGURE 2:**
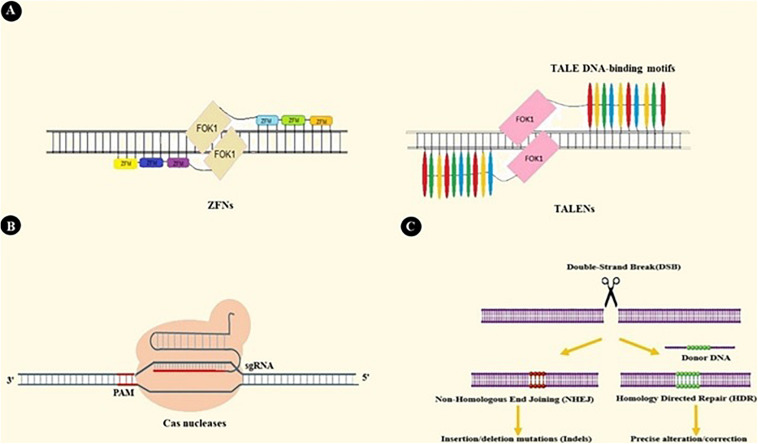
ZFNs, TALENs, and CRISPR-based genome editing. **(A)** ZFNs and TALENs are nucleases that operate based on protein-DNA interactions. Assembling Zinc Finger Motifs (ZFMs) and TALEs into larger complexes increases specificity. **(B)** CRISPR/Cas9 binds to DNA under the guidance of single guide RNA (sgRNA). sgRNA is a chimeric RNA constructed by fusing crRNA and tracrRNA to simplify the guidance system. As a result, Cas9-sgRNA is the most extensively used system in CRISPR based applications (23). For DNA recognition, many CRISPR systems also need Protospacer Adjacent Motif sequence (PAM) adjacent to the crRNA target site. PAM sequences are specific to each type of nuclease (e.g., NGG sequence is specific to SpCas) (21, 198). **(C)** Double-Stranded Breaks (DSBs) created by Cas9 in DNA structure activate two DNA repair pathways: Non-Homologous End Joining (NHEJ) and Homology-Directed Repair pathways (HDR). NHEJ results in random insertions or deletions at the target site, so it involves knocking out genes in CRISPR-based applications. HDR is a precise pathway that repairs target DNA breakage by using a homologous donor DNA. This pathway takes part in techniques that need more precise genome editing, like insertion or deletion of the desired DNA fragment (199, 200).

Up until now, three nuclease-based systems have been developed with ubiquitous genome editing applications and with the capacity to be engineered for specific sequence targeting. Zinc Finger Nucleases (ZFNs) are constructed by fusing zinc finger protein motifs with the DNA cleavage domain of FOK1 endonuclease. Zinc fingers are small protein motifs that can bind in the major groove of DNA in a sequence-specific manner. Multiple zinc finger modules can be assembled into a more massive complex to achieve higher specificity. As FOK1 needs homodimerization at the target site to cleave DNA, two separate zinc finger modules possessing adjacent target sites (Figure 2A) are incorporated. Generally, ZFNs possesses the potential to target sequences with 9–18 bp length (5–7).

Transcription Activator-Like Effector Nuclease (TALEN) is another nuclease-based system similar to ZFN in many aspects. TALENs are constructed by the chimeric fusion of the FOK1 cleavage domain to the complex of TALE DNA-binding modules. Each module is comprised of 34–35 amino acids with the ability to recognize a single base pair on the plus side. These TALE proteins, which were first discovered in Xanthomonas bacteria, can be designed to target 7–34 bp-long DNA targets (Figure 2A) (8–10).

The third system, CRISPR/Cas, was first introduced by Cong et al. and Mali et al. as a mammalian cell genome editing platform (11–13). This discovery has led to dramatic improvements in genetic manipulation specificity and efficacy. Moreover, it has expanded the application spectrum beyond mere genome editing, and new prospects of therapeutic and research purposes are being explored (14–17).

CRISPR/Cas constitute adoptive immunity against bacteriophages, transposable elements, and plasmids in Bacteria and Archaea. Upon pathogen invasion, these organisms insert segments of the invader genetic material into the CRISPR loci as new spacers within two repeat sequences. In cases of future invasions, these loci are transcribed into pre-crRNA and subsequently processed by Cas and other cellular agents into its mature form, CRISPR RNA (crRNA) (18–20). After that, the (crRNA)/Cas protein(s) complex detects mobile genetic elements with sequence specificity primarily arising from Watson-Crick base pairing between crRNA and target DNA (21).

There are two major classes and several types of CRISPR systems based on differences in components and mechanisms of action. In contrast to other classes, which rely on many effector proteins for RNA-guided target cleavage, the class 2 system relies on only one RNA guided endonuclease (Cas9 in type2 and Cpf1 in type5), turning it into a more straight-forward genetic-altering device. Thus, researchers have widely used this class of CRISPR system (14, 19–21).

CRISPR/Cas9 is the most extensively used CRISPR system in genome editing techniques. This system employs Cas9 as its RNA-guided endonuclease and crRNA as a guiding RNA, as well as trans-activating CRISPR RNA (tracrRNA). tracrRNA is a non-coding RNA and is critical for crRNA processing, Cas9 binding, and target DNA breaking (Figures 2B,C) (in the type5 CRISPR system, Cpf1 can detect and cleave the target DNA independently of tracrRNA) (22–24).

**FIGURE 3:**
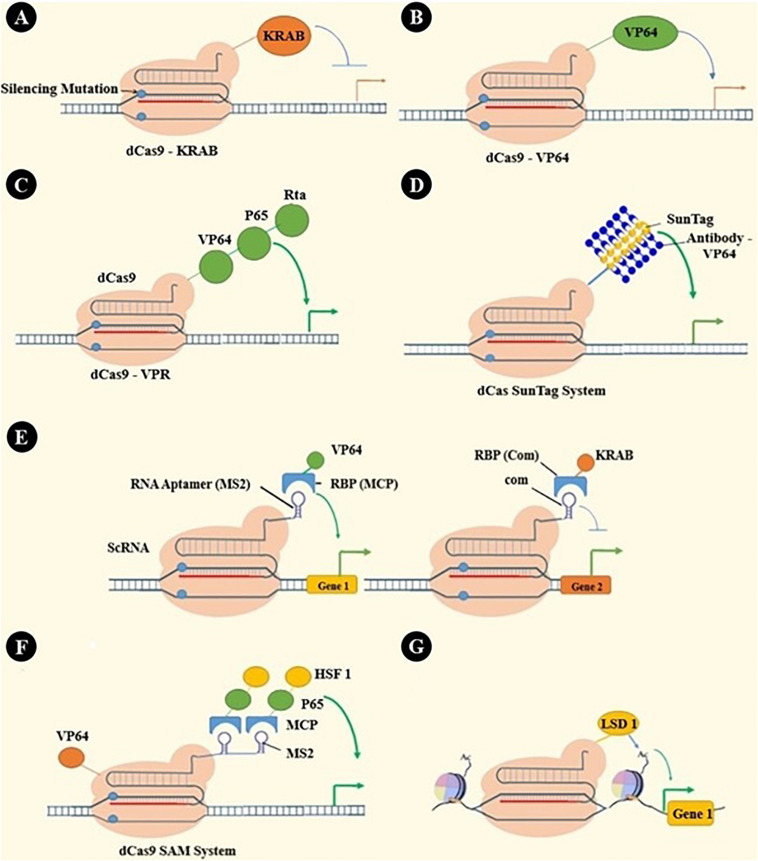
dCas applications: beyond genome editing. **(A)** dCas9-KRAB has been engineered by fusing the KRAB transcription repressor domain to dCas9 (201). **(B)** VP64, as a transcription activator, has been fused to dCas9 to activate a specific gene’s transcription (151, 152, 202, 203). **(C)** Recruiting multiple transcriptional activators shows synergistic effects that have led to engineering many systems, such as VPR by in-tandem fusing of both p65 and Rta to VP64 (204). **(D)** SunTag is a repeating protein-peptide array that recruits multiple antibody-fusion proteins. Protein domains, such as transcriptional activating or epigenetic modulating domains, can be recruited by antibody-mediated binding to SunTag, which is fused with dCas (205, 206). **(E)** RNA aptamers (e.g., MS2, com, PP7) can be fused with sgRNA to create a scaffold RNA (scRNA) that can recruit RNA-binding proteins (RBPs, e.g., MCP, Com, PCP). Fusing each RBP to the effector protein has enabled gene activation, repression, or even simultaneous activation and repression in one cell (207). **(F)** In the synergistic activation mediator (SAM) system, effectors are recruited by both dCas and scRNA (208). **(G)** Epigenetic modifying enzymes like P300 and LSD1 can be fused with dCas and alter cells’ epigenomic features. These alterations were locus-specific epigenetic editing, including histone modifications and DNA methylations (205, 209, 210).

Catalytically inactivated Cas9, dCas9, has many applications beyond genome editing. As dCas9 cannot break DNA strands, it is used as a sequence-specific DNA binding element. Through binding different effector parts to dCas9, it can be used as a transcription inhibitor or activator or even epigenetic modulator (16). These applications and some other applications are summarized in Figure 3.

### CRISPR Versus ZFN and TALEN

CRISPR has demonstrated considerable advantages over ZFNs and TALENs as it functions through DNA-RNA interaction. Since ZFN and TALEN rely on protein-DNA interaction for their sequence specificity, targeting a new site requires engineering a new protein, hence constraining these tools’ implementation in high-throughput applications. Additionally, the high molecular weights of these proteins in correspondence to the length of the sequence they target hinders multi-targeting during a single process of genetic engineering, which is mainly due to the genetic delivery limitations to the host and the metabolic burden they impose on the target cell (25). In contrast, the CRISPR system can be retargeted to a new site only by changing the guide RNA sequence (4, 21). Moreover, multiple site-specific genetic alterations are possible through the delivery of a single form of Cas and multiple sgRNAs requiring fewer macromolecules than multiple ZFNs and TALENs, which in turn results in lower cellular toxicity. Besides, some have declared that CRISPR possesses higher genome editing efficiency relative to ZFNs and TALENs (11, 26, 27).

## CRISPR/Cas and Tumor Cell Manipulation

Characterizing and targeting genes responsible for tumorigenesis and cancer progression remains crucial yet challenging. Alterations in regulations of these genes, dubbed as oncogenes and tumor suppressors, mediate resistance to therapy and cancer progression. The CRISPR/Cas system has been exploited for discovering and introducing such genes and their cognate pathways as novel targets and has served as a powerful tool for cancer gene therapy.

### Gene Therapy via CRISPR/Cas

The excessive accumulation of specific mutations leads to biological hallmarks of a malignant phenotype (28). Through gene therapy, CRISPR/Cas9 technology is applicable in oncogenes’ inactivation or restoration of tumor suppressors and apoptotic and immune-stimulatory functions.

A common characteristic of many tumor cells is a mutation in tumor protein 53 (*TP53* in human or *TrP53* in mice). This gene encodes the tumor suppressor protein p53, which is responsible for halting cell proliferation in response to internal stress and abnormality inputs (29). Almost half of human malignancies are harboring an altered form of TP53 (2). Albers et al. showed that CRISPR/Cas9-mediated inactivation of Transformation Related Protein 53 (TrP53) and expression of oncogene H-Ras led to cellular transformation and tumor formation in a xenograft model (30).

Restoring the mutated TP53 to its wild type function using various compounds can induce apoptosis and senescence in tumor cells. Chira et al. envisioned a novel Tp53 therapeutic concept, capable of replacing the entire mutant locus of TP53 (∼20.5 kb in length) with its functional cDNA version through homologous recombination. This recombination required the expression of two sgRNAs (single guide RNA comprising crRNA and tracrRNA fusion) binding to upstream and downstream flanking sites of the TP53 mutant locus. They designed a hybrid of an Adeno-Associated Virus and a bacterioPhage (AAVP) directed to tumor cells. Hence, the design increased the specificity, and it could also possess an inducible functionally through the administration of a simple antibiotic like doxycycline. The intravenous administration of this therapeutic vector yielded limited side effects and increased distribution, leading to sustained expression of p53 and tumor regression even in distant metastatic tumor sites (2).

Human Estrogen Receptor 2 (*HER2*) gene is a well-known oncogene and is over-expressed in some cancers, such as breast cancer, serving as a therapeutic target for Herceptin (Trastuzumab). As an alternative, Wang et al. utilized a novel strategy to target the oncogene *HER2* with the CRISPR/Cas9 system. Co-expression of Cas9 and three sgRNAs targeting *HER2* exons 5, 10, and 12 significantly reduced cell growth and tumorigenicity in Her2-positive breast cancer cells (31). One advantage of employing CRISPR/Cas9-mediated *HER2* down-regulation over conventional therapeutics such as monoclonal antibodies (mAbs) is the simplicity of designing new guide RNAs for targeting new mutations in the case of resistance. The development of conventional therapeutics would, on the other hand, require a new drug discovery program, which is a time-consuming and laborious practice.

Epidermal Growth Factor Receptor (EGFR) is a glycoprotein anchored to the cell’s membrane and has an intracellular tyrosine kinase domain. Constitutive tyrosine kinase activation due to genetic mutation causes cancer formation and progression. Although Tyrosine Kinase Inhibitors (TKIs) have been the therapeutic choice for EGFR-expressing malignancies, resistance against these medications develops within 2 years. Huibin et al. proposed a molecular surgery using the CRISPR system to repair the mutated EGFR using the CRISPR/Cas9 nickase platform. Alternatively, this strategy would halt its activity by introducing a stop codon or an indel (random insertions and deletions) through HDR and NHEJ, respectively (32). This approach offers personalized gene therapy for disease-causing genetic abnormalities, which can be coupled with traditional therapeutic strategies, including surgeries and radiotherapy.

One of the main approaches to cancer cell therapy is knocking out genes responsible for inducing drug resistance. NFE2L2 gene [i.e., encodes Nuclear Factor Erythroid 2-Related Factor (NRF2)] is up-regulated under various conditions, such as oxidative or electrophilic stresses. These are consequences of chemotherapeutic drug administration as well. NRF2 targets numerous genes encoding GSH mediators, antioxidant proteins, and efflux pumps and induces cells’ resistance against chemotherapy (33). Bialk et al. exploited CRISPR/Cas9 to knock out the NRF2 gene in chemo-resistant lung cancer cells. They reported restored effectiveness of anticancer drugs cisplatin, carboplatin, and vinorelbine post-gene editing (34). Therefore, the synergistic effects of combining gene edition and standard therapeutic options such as chemotherapy may address drug resistance-mediated refraction or relapse of the disease.

It is now known that epigenetic mechanisms play a critical role in different cancers’ formation and progression (35). Recently, the CRISPR/Cas9 system has shed light on the underlying epigenetic irregularities and rendered researchers able to target these irregularities using the CRISPR/Cas9 platform. Wang et al. (36) targeted granulin (GRN), a liver cancer stem cell marker, epigenetically using the CRISPR/Cas9 system. The system consisted of C-terminus of the catalytically inactive dCas9 fused to three epigenetic suppressor domains: DNMT3a, histone 3 K27 methyltransferase EZH2, and heterochromatin binding suppressor KRAB. The group then designed gRNAs specific to the GRN promoter. Epigenetic targeting of GRN decreased tumor cell growth compared with the random gRNA control and dCas9 control groups (36–38), thus introducing a powerful epigenetic tool for oncogenes’ inhibition.

Moreover, some viruses can cause malignant phenotypes in cells by inserting oncogenes into the cell genome. The CRISPR system can be used against these virus-encoded oncogenes as well. The most famous example is the human papillomavirus, which is a primary causative agent for cervical cancer (39). Hsu et al. targeted E6 and E7 HPV-encoded oncogenes with CRISPR/Cas9 in patient-derived xenografts of HPV16 + anal tumors in immunodeficient mice and showed growth inhibition in tumor cells (40). CRISPR/Cas9 also induced proliferation arrest and decreased viral load in patient-derived cells suffering from Burkitt’s lymphoma with latent Epstein-Barr virus infection (41). Based on these findings, CRISPR/Cas9 could be a promising therapeutic approach for the treatment of viral infection-related cancers.

As mentioned before, current CRISPR-based systems create DSBs which induce cellular DNA repair pathways, causing indels at the target locus. However, most diseases, including cancers, are caused by several point mutations, and there is a growing need for more efficient point mutation corrector tools. Therefore, novel Cas9 variants have been introduced that convert one base to another rather than creating DSBs. Accordingly, Komor et al. conjugated cytidine deaminase enzymes with dCas9 to convert a single base to another one (42). Their designed Base Editor (BE) was able to convert C to T to correct the p53 Tyr163Cys mutation that is associated with a variety of cancers (43). Following the same approach, Kuscu et al. introduced the CRISPR-STOP approach to create stop codons by converting single bases using BEs previously introduced by Komor et al. (44). Although the CRISPR-STOP approach has fewer potential sgRNAs for a target gene than wild type Cas9, introducing early stop codons is an efficient and safer gene-silencing approach.

### Prognostic and Predictive Biomarkers Identification via CRISPR/Cas

Cancer biomarker discovery has emerged as an exciting area of research in recent years, mainly due to advancements in investigational screening tools for molecular level signatures, such as genomic-based alterations. Biomarkers, which are objectively measured and assessed features as an indicator of a biological status or process, can play a pivotal role in a patient’s outcome. Prognostic and predictive biomarkers refer to biological characteristics that give information on the possible course of cancer and the patient’s overall outcome irrespective of the therapy and predict the potential therapeutic outcome of a targeted treatment. The CRISPR/Cas platform can serve as a valuable tool to identify these biomarkers though assessing genetic or epigenetic changes within the tumor tissue (45, 46).

Cluster of Differentiation 44 (CD44) is a cell surface molecule that interacts with hyaluronic acid (HA) in the extracellular matrix. This interaction activates the mitogen-activated protein kinases (MAPK) and PI3 kinases/akt pathways in the act of oncogenic pathways (47, 48). Studies have revealed that CD44 binds to P-glycoprotein (P-gp), which is encoded by the *ABCB1* gene and acts as a drug efflux pump (49). Hence, its overexpression leads to resistance against chemotherapeutic drugs such as vinblastine, doxorubicin, and paclitaxel in osteosarcoma cells (50, 51). Xiaoa et al. used CRISPR/Cas9 to knock out CD44 in drug-resistant cell lines. P-gp levels were decreased as a consequence of CD44 knockout. CD44 knockout diminished resistance to doxorubicin in osteosarcoma cells (52). This study revealed that CD44 expression could serve as a predictor for overall survival and chemotherapy response and shed light on its role in tumor migration and tumorigenesis.

The Wnt-signaling pathway is considered as a pathway that initially drives cells’ self-renewal in colorectal tumors (53). Mutations in this pathway induce the pathway to be constitutively active and provoke drug resistance. Furthermore, *TIAM1*, which encodes a guanine nucleotide exchange factor specific to Rac1, is a responsive gene to the Wnt signaling pathway and is overexpressed in human colon cancer (54). Izumi et al. generated xenograft mouse models with stable knockdown of *TIAM1* using CRISPR/Cas9 and subsequently treated them with 5-fluorouracil (5-FU). *TIAM1* knocked-down cells were more sensitive to 5-FU. Also, the tumor size and weight notably diminished compared to the controls. This study revealed the correlation between *TIAM1* overexpression in CRC cells and also cancer-associated fibroblasts with drug resistance, serving as a predictive tool, and introduced this molecule as a potential therapeutic target to reverse drug resistance (55).

Qian et al. evaluated the effects of targeted *DERARE* methylation on leukemogenesis. Distal Element Multiple Retinoic Acid Response Element (DERARE) is a specific Cis-Regulatory Element (CRE) that maintains the homeostasis between self-renewal and multi-lineage differentiation. This effect is due to the regulation of Hoxb cluster genes in a methylation-dependent manner, which prevents leukemogenesis. The CRISPR/Cas9 epigenetic editing tool employed in this study was constructed by fusing the DNA methyltransferase 3A (DNMT3A) catalytic domain to the C terminus of deactivated Cas9 (dCas9). In this fusion, there was a short linker enabling DNA methylation adjacent to the single guide RNA (sgRNA) binding site on human *DERARE* (56). The group infected human AML cell lines carrying the DNMT3A mutation via the lentiviral vector and observed a remarkable reduction in colony size and number in DNMT3A-dCAS9-treated AML cells (57). Their research proposes DNA methylation patterns on *DERARE* as a screening protocol for drug selection and implementing personalized therapeutic approaches.

MicroRNAs (miRNAs) are small, non-coding RNA molecules that play a role in cancer pathogenesis. There are different miRNAs implicated in all stages of cancer functioning as oncogenes or, conversely, as tumor suppressors. Hence assessing their upregulation and downregulation could be a potent tool to assess cancer progression (58). For example, a miRNA that is overexpressed in hepatocellular carcinoma cells is miR 3188. Zhou et al. demonstrated that this overexpression induces tumor formation and progression, resulting in poor clinical outcomes. Zhou and colleagues reported suppressed cell growth, colony formation, cell cycle progression, and increased apoptosis through CRISPR/Cas9-mediated miR-3188 knockout in cells (59), and proposed miR-3188 as a potent therapeutic target and also as a biomarker for early detection of HBV-related hepatocarcinogenesis among patients with a family history of HCC.

### Identifying Novel Targets and Deciphering Drug Resistance via CRISPR/Cas

CRISPR/Cas9 is a potent genome editing tool for discovering novel pathways and targets for cancer treatment and also unraveling the mechanisms responsible for inducing drug resistance and genes underlying these phenomena. CRISPR has an essential advantage over other genome editing technics (e.g., ZFNs and TALENs) because it can be easily retargeted to another locus in the genome by changing sgRNA design. Therefore, it is possible to knock out different genes with CRISPR/Cas9 in order to make explicit their role in cancer cell proliferation, survival, and metastasis, and also to dissect their contribution to developing drug resistance. Herein, we will review the results of some recent studies deploying this approach using the CRISPR/Cas9 genome -editing tool.

Feng et al. carried out an intervention using CRISPR/Cas9 to knock out *CDK11B* in osteosarcoma cells. Inhibiting *CDK11B* expression decreased cells’ viability and their migratory and invasive activity. This study revealed the *CDK11B* role in cancer pathogenesis and proposed its targeting as a potential approach in augmenting osteosarcoma patients’ survival (60).

Urokinase Plasminogen Activator (uPA) binds to its receptor (uPAR), which is encoded by the *PLAUR* gene. Their interaction gives rise to extracellular matrix remodeling and subsequent cell adhesion, migration, proliferation, and survival via various signaling pathways (61, 62). Wang et al. revealed that uPAR knockout by CRISPR/Cas9 reduces resistance to chemotherapeutical drugs such as 5-FU, cisplatin, docetaxel, and doxorubicin (63). Hence, this proves the uPA/uPAR pathway as a potential target for intervention by other agents as well.

G-Protein-Coupled Receptor (GPCR) constitutes 40% of all drug targets approved by the FDA. *GPRC5a*, which encodes the G-Protein-Coupled Receptor family C, member 5, group A, also named retinoic acid-inducible 3 (RAI3), is overexpressed in a variety of cancers, including pancreas cancer (64, 65), which makes it a potential biomarker for early diagnosis of cancer. Liu et al. investigated the resistance of *GPRC5a*-knockout cells against commonly used chemotherapeutic drugs such as 5-FU, gemcitabine, and oxaliplatin. The group observed suppressed resistance in *GPRC5a* knockout cells compared with wild type cells using the EC50 (Concentration for 50% of maximal effect) assay (66). Targeting this signaling pathway, thus, appears to be an exciting area of further research.

*KRAS* is the most frequently mutated proto-oncogene in cancer cells, and its pharmacological targeting has remained challenging in cancer therapy (67). Since activating mutations in *KRAS* are a hallmark of pancreatic ductal adenocarcinoma (PDAC), CRISPR/Cas was employed in a study to model complete *KRAS* inhibition and predict resistance mechanisms in a subset of human and mouse PDAC cells. This study showed the merit of *KRAS*-directed therapies in reducing *in vitro* proliferation and *in vivo* tumorigenic growth and revealed *KRAS*’ role in balancing proliferation and metastasis in tumor cells. This study also recommended PI3K pathway activation as a potential resistance mechanism in *KRAS* knocked-out cells and suggested simultaneous PI3K and *KRAS* inhibition as a therapeutic strategy for PDAC (68).

The N-Myc oncoprotein, which is overexpressed in a fraction of different types of prostate cancers (69–71), possesses identified functions in tumor progression (70, 72). Yin et al. reported that the N-Myc-regulated DNA Damage Response (DDR) pathway (N-Myc/miR-421/ATM) is associated with tumor progression and hormonal therapy resistance, such as enzalutamide resistance. N-Myc overexpression cooperates with EZH2 to suppress miR-421. This suppression leads to ATM up-regulation, resulting in enzalutamide resistance. Yin and colleagues knocked out ATM via the CRISPR/Cas9 genome editing tool and detected re-sensitized prostate cancer cells (73).

Apolipoprotein B mRNA editing the enzyme catalytic polypeptide-like (APOBEC) enzyme family is involved in genetic instability and heterogeneity. APOBEC enzyme family derived DNA mutagenesis patterns have been detected in different types of cancers (74), including breast (75), bladder, cervix, head and neck, lung cancers (76), and gliomas (77). Schmitt et al. investigated the role of APOBEC3B in glioma cells’ resistance to temozolomide by determining the caspase 3/7 activity. They knocked down APOBEC3B using CRISPER/Cas9 and observed higher sensitivity toward temozolomide relative to the control cells (77).

miR-21 is a miRNA overexpressed in cancers. Huo et al. hampered its expression via indels introduced by CRISPR/Cas9. The group reported the inhibition of cell proliferation, migration, and invasion in ovarian cancer cells. The hampered expression of miR-21 was associated with higher drug sensitivity and decreased Epithelial to Mesenchymal Transition (EMT), revealing its role in tumor metastasis (78). The latter plays a critical role in metastasis and chemoresistance (79).

### CRISPR/Cas Genome-Wide Knockout Screening

CRISPR/Cas genome-scale knockout screening refers to the process of sgRNA-mediated disruption of genes’ functionalities aiming to discover novel genes and pathways underlying various phenotypes and biological processes, including tumorigenesis and drug resistance in cancer, and also their potential as therapeutic targets (80, 81). Numerous studies have employed genome-wide knockout libraries (GeCKO) which contain a multitude of sgRNAs against a defined set of genes involved in cancer. sgRNAs in the cells affect their competence for viability during the proliferation. The enrichment or depletion of these sgRNAs thereupon reveals the genes responsible for the cognate phenotype (82–88).

Among the pioneering groups to both develop and implement GeCKO (15, 89), Shalem et al. unraveled novel genes responsible for the development of resistance to Vemurafenib (PLX) (i.e., a BRAF protein kinase inhibitor) in melanoma. Cells were transduced with a pool of lentiviruses each carrying Cas9 and a sgRNA. Thereafter, cells underwent PLX selection. The enriched sgRNAs in viable cells were then sequenced and their target genes, whose loss of function contributed to the development of resistance, were revealed (15). Decoding the mechanisms underlying resistance to drugs can introduce novel predictive biomarkers and even novel targets.

In a similar study, Manguso et al. unraveled the mechanisms underlying the resistance to PD-1/PD-L1 inhibition treatment using a library of lenti-vectors with sgRNAs targeting 2368 murine genes. This study revealed Ptpn2 as a potential target to revert PD-L1 immunotherapy resistance since its knockout was associated with increased sensitivity to immunotherapy (90). IRF4, STAT3, SOS1, and GRB2 genes’ knockout in ALK + ALCL cells also attenuated PD-L1 expression and undermined PD-L1 mediated T cells and NK cells suppression (91).

Genes responsible for the resistance to Bortezomib (BTZ) in Multiple Myeloma have been determined by conducting a genome-scale positive selection assay. Multiple myeloma cells transduced with sgRNA-carrying lentiviruses were cultured in the presence of BTZ at its lethal dose. The inactivated genes in survived cells were identified based on the enriched sgRNAs sequencing, and proteasome regulatory subunit PSMC6 was proven to be the only gene that granted resistance to BTZ reproducibly. Therefore, PSMC6 emerged to be a promising predictive biomarker and also a novel target (92).

This strategy has also been employed to discover the mechanism of action of Immunomodulatory imide drugs (IMiDs), including mediating pomalidomide and lenalidomide. Liu et al. explored the mechanism responsible for the susceptibility of multiple myeloma cell lines to IMiDs by loss-of-function genome-wide screening. The team found CRBN regulation mediated by CSNs as the major factor determining multiple myeloma cells’ sensitivity to IMiDs (93).

CRISPR genome-wide screening has also proven to be beneficial in novel target identification. A dropout screen on AML cell lines revealed that the sgRNA-mediated knockout of KAT2A hampers AML cell lines’ growth. The same outcome was observed when AML cell lines were treated with MB-3, a KAT2A inhibitor. Therefore, since KAT2A is not an essential gene for hematopoietic progenitor cells, its inhibition is proposed as a novel anti-AML therapeutic strategy and MB-3 as a potential medication for AML (94).

Another genome-wide negative selection screen on AML employed mouse lentivirus-based GeCKO v2 library. Among the genes dispensable for human hematopoiesis, the mRNA de-capping enzyme scavenger (*DCPS)* appeared to be essential for AML cell survival. Its inhibitor, RG3039, exhibited anti-leukemia effects in human AML xenograft models, sparking its combination with other drugs as a potential therapeutic approach (95).

CRISPR/Cas has surpassed RNA interference (RNAi) technology in building genome-wide knockout libraries due to having a lower rate of off-targets and also introducing loss-of-function mutations into the gene’s sequence as opposed to RNAi which only yields the gene’s partial suppression in the majority of cases (15, 81, 96). However, CRISPR/Cas imposes some limitations; first, during drop-out screenings, CRISPR/Cas has shown conditional false-positive results in cancers with aneuploidy. Second, genomic regions with multiple copy numbers, including non-expressed genes, are subject to excessive double-strand breaks (DSBs). DSBs in turn lead to substantial DNA damage and subsequently induce apoptosis. Hence, sgRNAs targeting non-expressed genes should be excluded from the libraries. Third, sgRNAs are conventionally designed to target 5’ exon. However, false-negative results have been attributed to genes with initiation points in other exons as well, signifying the effect of sgRNA positioning in the accuracy of the outcomes (97).

## CRISPR and Immunotherapy

It has been established that evading immune destruction is one of the hallmarks of cancer. Tumor cells can inhibit immune effector cells or cause immune tolerance through the secretion of extrinsic factors affecting the tumor microenvironment (TME) (98). Among all the immune system members present at TME, macrophages and T cells are the most distorted. Tumor-associated macrophages (TAMs) support tumorigenesis and metastasis and inhibit antitumor responses by releasing EGF, IL-6, TNF, MMPs, VEGFA, TGF-β, IL-10, and PD-L1 (98, 99). In addition, T cells’ anti-tumor activity and metabolic state is disrupted by the immune-modulatory cytokines present in the TME and the immune checkpoint inhibitors such as PD-1 and CTLA-4 (99, 100). Accordingly, a study by Chung et al. on 11 breast cancer cases revealed that the presence of M2 macrophages in the TME was correlated with T cell exhaustion (101). Therefore, the state of TME strongly affects the patients’ prognosis. Hence, identifying the mechanisms underlying the tumorigenic characteristics of interactions between immune suppressive cells and tumors can reveal novel therapeutic targets for developing antagonists, such as mAbs, and immuno-modulatory drugs intervention.

Another approach is to fortify already existing immune responses or develop new ones through bypassing their dependence on the robust and intact immune system, which, as explained before, had transformed into a non-functional state (102). Adoptive T cell immunotherapy of cancer has recently proven its potential in numerous clinical trials, yet it still suffers from various predicaments (103).

CRISPR/Cas-mediated genetic manipulation has strived to address some of the challenges mentioned above regarding immune system misfunctioning from various prospects, some of which are discussed below.

### Novel Immune System-Tumor Interplay Identification via CRISPR/Cas

TME is composed of tumor cells, stromal cells, and immune cells, and the interaction among these cells affects tumor progression. Stimulants present in the TME, such as cytokines, chemokines, and growth factors, determine the polarization of macrophages and their differentiation toward M1 or M2 subtypes. M1 macrophages are able to: (i) release pro-inflammatory cytokines such as IL-12, IFN gamma, IL-1, IL-23, and iNOS; (ii) reeducate the DC and CD4 + T cells; and (iii) activate CD8 + T cells and, as a result, promote an immune response against the tumor and prevent tumor progression. In contrast, M2 macrophages and TAM increase angiogenesis and formation of tumor-associated fibroblasts. These cells attenuate immune responses in the TME and increase tumor progression (104–107).

Various agents that participate in the interaction between tumor cells and M2 macrophages have been targeted using the CRISPR/Cas9 system. These targets include: (I) macrophage Signal Regulatory Protein a (SIRPa). The crosstalk between SIRP-a on macrophages and CD47 receptors on tumor cells prevents phagocytosis of cancerous cells via the “Don’t eat me” signal. Turning off this signaling by knocking out the SIRP-α using CRISPR/Cas9 enables phagocytosis of cancer cells (108); (II) Kindlin2, this protein is another therapeutic target that increases the secretion of Cancer Stimulating Factor1 (CSF1) from tumor cells. Moreover, Kindlin2 induces chemotaxis of macrophages to the TME, which subsequently constitutes the dominant population. Ablating Kindlin2 expression using CRISPR/Cas9 technology inhibited invasion and migration of tumor cells without affecting their proliferation rate (109); (III) Osteopontin (OPN) glycophosphoprotein in tumor cells increases the recruitment of M2 macrophages. Therefore, it has been targeted in cancer treatment. OPN knockout in tumor cells by CRISPR/Cas9 decreases the chemotaxis of macrophages and increases the sensitivity of cancer cells to CD8 + T cells cytotoxicity (110); (IV) Lysosome Associated Membrane Protein Type 2A (LAMP2a) is another agent expression which is increased by tumor cells in TAMs. LAMP2a inactivation using CRISPR/Cas9 reduces TAM activation and prevents the suppression of the immune system and decreases tumor growth (111); (V) IL-8 released by macrophages increases tumor growth and metastasis. It has been revealed that knock out of the IL-8 receptor, CXCR2, by CRISPR/Cas9 in triple-negative breast cancer cell lines reduces the progression of the tumor (112); and (VI) Tumor-Secreted Protein S (Pros1) is the best-studied ligand of Tyro3/Axl/Mer (TAM) receptor tyrosine kinases, and its CRISPR-based deletion inhibits M2 polarization, leading to heightened immune infiltration and reduced tumor viability (113).

Theoretically, many other potential targets can affect macrophage polarization toward the M2 phenotype and their immunosuppressive features. Therefore, this could be a hot topic for future investigations not only in treating cancer using CRISPR technology but also in finding suitable targets for pharmacological drugs or monoclonal antibodies.

### Novel Cellular Immunotherapy via CRISPR/Cas

Immune cell therapy has emerged as a novel approach after traditional pharmaceuticals such as small molecules and biopharmaceuticals, like therapeutic proteins, including mAbs (114). Due to an extensive TCR repertoire and their ability to distinguish themselves from non-self-epitopes produced during tumorigenesis, T lymphocytes play a pivotal role in tumor surveillance and cancer eradication. Thus, attempts have been made to produce, guide, or enhance cellular immunotherapy against cancer over the past decades. T cell-based immunotherapy is attributed to the implementation of *ex vivo* manipulated T lymphocytes aiming to eliminate tumors with TCR-engineered T lymphocytes and Chimeric Antigen Receptors T cells (CAR T Cells) as its main strategies (83, 102). In the former, patient’s T lymphocytes are transfected to express a transgenic TCR derived from other patients or animal models with specificity against a Tumor-Associated Antigen (TAA), and the latter exploits chimeric receptors with their antigen recognizing domains mostly acquired from antibodies (115).

#### Universal CAR T Cells Production via CRISPR/Cas

Despite significant breakthroughs in CAR T Cells’ application in hematologic malignancies elimination, there are still considerable barriers in the application of CAR T cell therapy, specifically against solid tumors, and also throughout its laborious manufacturing process (116). In CAR T cell therapy, T cells can be derived from patients (autologous) or an allogeneic donor. Using autologous T cells is a time-consuming process and largely depends on the quality and quantity of autologous T cells harvested from the patient. These issues are also coupled with the expense of manufacturing autologous T cells (117, 118). One of the substantial barriers in using allogeneic T cells is the presence of endogenous MHC class I and TCR on donor’s T lymphocytes, which cause alloreactivity and graft-versus-host disease (GVHD), respectively. The former is dependent on the TCR repertoire of the recipient (119, 120).

To overcome the aforementioned HLA barriers in the implementation of third-party donors-derived T lymphocytes, Poirot et al. were first able to knock out endogenous TCR by TALEN-mediated disruption of T cell receptor alpha constant chain (TRAC) in lentiviral-transduced CD19 CAR T cells (121). This platform was further entered into clinical studies on two infants with relapsed refractory CD19 + B cell ALL (122). “Universal CAR T cells” are now being manufactured by knocking out TCR and HLA-I in allogeneic T cells (123). Furthermore, Eyquem et al. have exploited CRISPR/Cas9 to insert the CAR gene and remove the TCR gene concurrently by introducing the CAR gene into the TRAC locus. They observed a regular CAR expression in T cells, increased potency of T cells, and decreased terminal differentiation and exhaustion in the mouse model of AML (124).

CRISPR/Cas9 technology was further used to couple allogenic CAR T cells and checkpoint pathway disruption. In a study by Ren et al. CRISPR/Cas9 technology was employed to knock out PDCD1, TRAC, and beta-2-microglobulin (β2M), which encodes the accessory chain of MHC class I in CD19 or PSCA CAR T cells. These T cells exhibited robust antitumor activity and did not induce GVHD in the leukemia mice model (125). In a similar study on EGFRvIII-targeted CAR T cells and their triple gene-edited CAR, T cells displayed an enhanced profile in preclinical glioblastoma models (126).

Of T cell-based immunotherapies, the necessity of TCR knockout is not confined to CAR T cell therapy. In TCR-engineered T cells, the α and β chains of endogenous TCR are shown to pair with the transgenic TCR α and β chains. This mispairing disrupts efficient T cell redirection toward the targeted antigen and also poses the risk of novel autoreactivity. Moreover, competition of these four chains for binding to the CD3 complex hampers the translocation and, consequently, the sufficient display of transgenic TCR on the cell surface. These are among the culprits underlying the poor performance of TCR-engineered T cells in clinical studies (127, 128). In a pioneering study by Provasi et al., ZFNs were designed to obstruct endogenous TCR β and α chain genes expression and a higher level of cell surface exogenous TCR display alongside superior specificity was reported (129). CRISPR/Cas-mediated disruption of surface TCR expression in TCR-engineered T cells has paved its way into clinical studies, and will be discussed in the following sections (130).

#### Immune Checkpoint Inhibition via CRISPR/Cas

Engineered T cells’ activity is susceptible to be impeded via natural immune checkpoint regulators. Thus, identification of these immune checkpoint regulators, such as programmed cell death protein 1 (PD-1), cytotoxic T-lymphocyte-associated protein 4 (CTLA-4), and other inhibitory signaling, has created a new vision for cancer immunotherapy (131, 132). Overexpression of immune checkpoint regulators and up-regulation of their cognate inhibitory ligands (e.g., PDL1 and CTLA4 ligand) in the TME may limit TCR-engineered and CAR T cell persistence and function. Accordingly, this will lead to impaired clinical outcomes of this strategy (15). The CRISPR/Cas9-based editing could be used to ablate PD-1 and CTLA-4 in order to increase the efficiency of T cell-based immunotherapy (125).

Gene knock-out of PD-1 in Car T cells using CRISPR technology was first applied by Su et al., and resulted in enhanced cytotoxicity without affecting T cells viability (133). Since then, several groups reported similar results while administering different methods. Hu et al. used CRISPR/Cas9 against the *PDCD1* gene, accompanied by anti CD133 CAR insertion into the genome using the piggyback transposon system. They reported increased T cell proliferation and cytokine secretion (134). In another study, a ground-breaking one-step system named knock-in and immune-checkpoint knockout (KIKO CAR-T cell) was developed, which relied on the cpfl system to mediate simultaneous knock-in of two different CARs and knockout of PD-1 and TRAC. Elevated cytokine production and cytotoxicity and decreased levels of exhaustion markers were reported (135). PD-1 knockout CAR T cells were also assessed against glioblastoma, hepatocellular, and K562 tumor cell lines and demonstrated enhanced anti-tumor activity, reduced exhaustion, and augmented killing power in Car T cells (136–138). CRISPR/Cas9-mediated PD-1 knockout in T cells is now under clinical evaluation in a phase one trial on metastatic non-small-cell lung cancer (139).

Zhang et al. successfully generated lymphocyte activating gene-3 (LAG-3) knock-out CAR T cells using CRISPR/Cas9. They reported no significant viability or immunophenotypic changes in cultured CAR T cells *in vitro*. However, LAG-3 knockout CAR T cells possessed more vigorous antigen-specific anti-tumor activity in a xenograft mouse model (140).

The Fas receptor CD95 binds to its ligand, which is overexpressed on tumor cells and induces T cells’ apoptosis and loss of function (141). In order to increase resistance to Fas-mediated apoptosis, Ren and colleagues generated allogeneic universal CAR T cells via double knockout of endogenous TCR and HLA class I (HLA-I). Afterward, they disrupted the Fas receptor, PD1, and CTLA-4 in the same way and finally accomplished quadruple gene disruption in T cells with the one-shot system. Also, generating universal allogeneic T cells with multiple negative regulators (i.e., PD-1 and CD95/Fas death receptor) knocked out has been demonstrated (142).

#### CAR T Cell Functionality Augmentation via CRISPR/Cas

Using CRISPR/Cas9 to knock out the Granulocyte-Macrophage Colony Stimulating Factor (GM-CSF) gene in CAR T cells increased their antitumor activity and survival. Knocking out the GM-CSF gene not only increased CAR T cell activity but also decreased neuroinflammation and the probability of CRS (143).

It is known that CD7 targeting CAR T cells can destroy each other by targeting CD7 markers present on themselves, an action termed fratricidal activity. Silva et al. indicated that knocking out CD7 in CAR T cells using CRISPR/Cas9 prevents fratricidal activity followed by CAR T cell immunotherapies (144). Moreover, knocking out CD7 and TRAC in CAR T cells increased the efficacy in the treatment of T cell acute lymphoblastic leukemia (T ALL) (145).

Jung et al. used CRISPR/Cas9 to knock out Diacylglycerol Kinase (DGK) in CAR T cells. DGK is an enzyme that metabolizes diacylglycerol to phosphatidic acid (PA), knocking out the DGK gene increases CD3 signaling and improves T cell function by boosting TCR signaling (146).

Studies have confirmed that knocking down *TET2*, a tumor suppressor gene, leads to epigenetic and phenotypic alterations in T cells, which can improve clinical results (147). Fraietta et al. showed that using CRISPR/Cas9 to knock out the CD19 CAR gene into the *TET2* locus promotes anti CD19 CAR T cell activity (147).

### CRISPR/Cas and Oncolytic Virotherapy

Oncolytic viruses (OVs) possess a higher tendency to infect and replicate within cancerous cells than normal cells. Based on the promise of naturally occurring OVs, their further genetic manipulation has turned into an up-and-coming strategy (148). These manipulations mainly focus on increasing their tumor-selectivity, tumor tropism, and therapeutic efficacy and decreasing their off-tumor toxicity and pathogenicity against non-neoplastic cells. They mainly stem from the normal cells’ ability to develop intracellular anti-virus defense mechanisms which are mostly accompanied by the host cell’s apoptosis. In neoplastic cells, on the other hand, anti-apoptotic molecules are overexpressed and programmed cell death is obscured. Hence, the anti-virus immune-compromised cells turn into a suitable place for OVs to replicate and subsequently to burst the cell itself and to spread viral particles’ locally among other cancerous cells (149, 150). The first and only OV to attain FDA approval is T-VEC (IMLYGIC^®^) with an indication for advanced melanoma (151). T-VCE is an attenuated Herpes Simplex Virus 1 (HSV-1) with decreased pathogenicity and increased tumor selectivity. It is also engineered to release GM-CSF and to enhance MHC-mediated antigen presentation (151). The potential of OV in cancer therapy and its genetic manipulation by strategies except CRISPR/Cas have been extensively reviewed elsewhere (152–155). Here, the focus is on the application of CRISPR/Cas in oncolytic virotherapy.

Genetic manipulation of OVs with large genomes, such as HSV, Adenovirus (Adv), and Vaccinia Virus (VACV), by traditional techniques is laborious and has low efficiency. CRISPR/Cas, on the other hand, expedites recombinant OV generation. It simplifies the process of deletion and insertion compared to the traditional strategies (156).

To achieve tumor selectivity and oncolytic efficacy, HSV-1 genes have been repeatedly subject to CRISPR/Cas-mediated knockout both through NHEJ (156) and HDR-mediated replacement with foreign genes (156–158). CRISPR/Cas has also been able to mediate multiple knockouts in HSV-1 (158). Various approaches have been implemented to further enhance the knockout efficiency, including enrichment via selectable markers, and also the incorporation of Scr7, an NHEJ inhibitor, to enhance HDR/NHEJ ratio in HSV-1 (157, 158). Collectively, all of these studies revealed low off-target activity of the CRISPR/Cas platform (156–158). HDR-mediated knock-in has demonstrated to be feasible and more efficient relative to mere homologous recombination in HSV-1 in several studies (156–158).

VACV is another OV with great therapeutic potential with numerous complete and undergoing clinical trials (159). CRISPR/Cas brought about simultaneous knockout of viral N1L, which plays a pivotal role in VACV virulence and host immune response modulation to VACV and TRP2, which is a TAA, inside the N1L locus (160). Theoretically, delivery of TRP2 to the tumor and induction of adaptive immune response can promote VACV into a cancer therapeutic viral vaccine. CRISPR/Cas-mediated simultaneous double knockout of two immune-regulatory genes, N1L and A46R, has also been reported and is presumed to enhance VACV immune response induction. Accordingly, it can be concluded that sgRNA-guided Cas9 can concomitantly target multiple sites on the VACV genome (160).

CRISPR/Cas system was used to exert indels into the EGFP gene in a recombinant adenoviral vector. The mutations’ inheritance to the next generations alongside its safety in terms of off-tumor activity were observed as well (156).

Plus, the CRISPR/Cas platform can be utilized to equip OVs with immune-stimulants or anti-tumoral agents. In a study by Cai and colleagues, oncolytic Human Simplex Virus 2 (oHSV-2) was armed with murine IL-15 via CRISPR/Cas9. The continuous intra-tumoral expression of IL-15 enhanced T cells anti-tumor response and also reduced the tumor mass. Incorporation of immune-modulators within the genome of OVs obviates the need for their systematic administration. Due to the local release of the immune-stimulants, this strategy subsequently reduces the risk of systematic side effects (161).

In addition, OVs can join their gene therapy delivery potential and their intrinsic anti-tumor activity to elicit a synergistic therapeutic response. Following the primary finding that CRISPR/Cas-mediated RAS knockout results in tumor regression in Rhabdomyosarcoma, Phelps and colleagues developed a CRISPR/Cas-harboring recombinant Myxoma Virus. This OV carried a spCas9-2A-Csy4 cassette followed by two NRAS-targeting sgRNAs. Csy4 ribonuclease was incorporated to split the sgRNAs from the 3’ end of mRNA molecules. A significant although not sustainable reduction in xenograft tumor growth was reported and further enhancing the tumor-specificity of this OV is recommended (162).

## CRISPR/Cas in Cancer Clinical Trials

In early 2018, the University of Pennsylvania, in collaboration with Tmunity, launched the first-in-human phase 1 CRISPR gene editing trial on NY-ESO-1 targeting TCR-engineered T cells in melanoma and myeloma patients. These T cells lacked TRAC, TRBC, and PDCD1 genes expression through CRISPR/Cas9-mediated editing. This study confirmed the safety of multiplex CRISPR-Cas9 editing of the human genome since neither of the subjects displayed cytokine release syndrome (CRS) or other side effects. Additionally, no rejection of transferred T cells due to pre-existing immunity against Cas9 among individuals was observed, further approving the potential of this technology in cancer treatment (130).

While Allogene Therapeutics and Cellectis utilize TALEN to produce TCR and MHC knockout universal CARs in their clinical trials (NCT04093596, NCT04106076, NCT03190278 to name a few), in July 2019, CRISPR Therapeutics initiated its first clinical trial evaluating CTX110 (NCT04035434). CTX110 are universal CD19-directed CAR T cells developed through using CRISPR/Cas9 to insert CAR into the TRAC locus, resulting in endogenous TCR disruption and also knockout of B2M and thus MHC1 for Refractory/Relapsed B cell malignancies.

In a clinical trial by Baylor College of Medicine (NCT03690011), T cells underwent endogenous CD7 knockout through CRISPR/Cas9 technology prior to receiving CD7-targeting CARs in order to avoid fratricidal activity. This study, which is designed for high-risk T-cell malignancies, is yet to recruit patients.

The Clinical application of the CRISPR/Cas9 platform is not confined to engineering cellular-based therapeutics. In a study initiated in mid-2018 (NCT03606486), the University of Washington developed a minimally invasive test to detect ovarian cancer through screening cervix pap smear samples for tumor-associated mutations in TP53 (i.e., the most common mutated gene in ovarian cancer). The team employed CRISPR-Duplex sequencing, which combines ultra-accurate Duplex Sequencing with CRISPR/Cas9 excision of target regions, leading to enrichment through size selection before sequencing library preparation (163). In another study in the Children’s Research Institute (NCT03332030), an induced Pluripotent Stem Cell (iPSC) bank was established for patients with Neurofibromatosis type 1 (NF1) phenotype. After that, CRISPR/Cas9 technology was utilized to develop isogenic NF1 wild-type (NF1 + / +), NF1 heterozygous (NF1 ±), and NF1 homozygous (NF1-/-) iPSC lines from individual patients. These iPSC lines are then differentiated to central nervous system tumor-relevant cells. They are screened to identify the drugs that have the potential to reverse or alleviate the disease phenotypes.

## CRISPR: Limitations to Solve

CRISPR immune-related adverse effects pose constraints in its therapeutic applications. The delivery system, Cas protein, and sgRNA can all evoke the host innate and acquired immune system. Pre-existing antibodies against SaCas9 and SpCas9 were found in 78 and 58% of donors, respectively. Likewise, anti-SaCas9 and anti-SpCas9 T cells were found in 78 and 67% of donors (164). Furthermore, pattern recognition receptors might recognize the secondary structure of sgRNAs and initiate an immune response (78). Further studies are required to ascertain whether or not these immune reactions could restrict the clinical potential of this platform (4).

The CRISPR/Cas9 system creates double-strand breaks in the genome, which can induce p53-mediated apoptosis in transfected cells and reduce cell viability. Wildtype p53 cells may die, whereas mutant p53 cells can be selected as a result of Darwinian selection-like evolution. Therefore, P53 inhibition can improve the efficacy of genome editing. In order to reduce the risk concerning p53 mutation in cells in cell replacement therapies, p53 function should be monitored (165, 166).

At the CRISPR target site, significant on-target mutagenesis, such as deletions and genomic rearrangements, were reported, which may have pathogenic effects (167). Incorporating gene encoding chimeric suicide receptors alongside the platform can induce drug-dependent apoptosis. These receptors bind to a drug by their extracellular domain and use a caspase9 endodomain as an effector (168). This strategy and similar common strategies in CAR T cell therapy using the suicide gene increases safety in using CRISPR mediated genome-edited cells in case of unpredicted adverse effects (169).

Off-target effects (OTEs) are still the primary limiting concern in the application of genome editing in clinical trials (170). Designing appropriate gRNA, selecting more specific nucleases, and restricting the exposure time to active nucleases are three primary strategies in reducing OTEs.

Appropriate gRNA design can significantly reduce OTEs and many gRNA design software tools that predict the probability of off-target cutting, such as *elevation*, *azimuth*, and *benching* (171). Truncating 5′ end of gRNAs and chemical modification of crRNA sites for impairing hybridization to off-target sequences are possible changes implicated in gRNA that improve specificity (172, 173).

**FIGURE 4:**
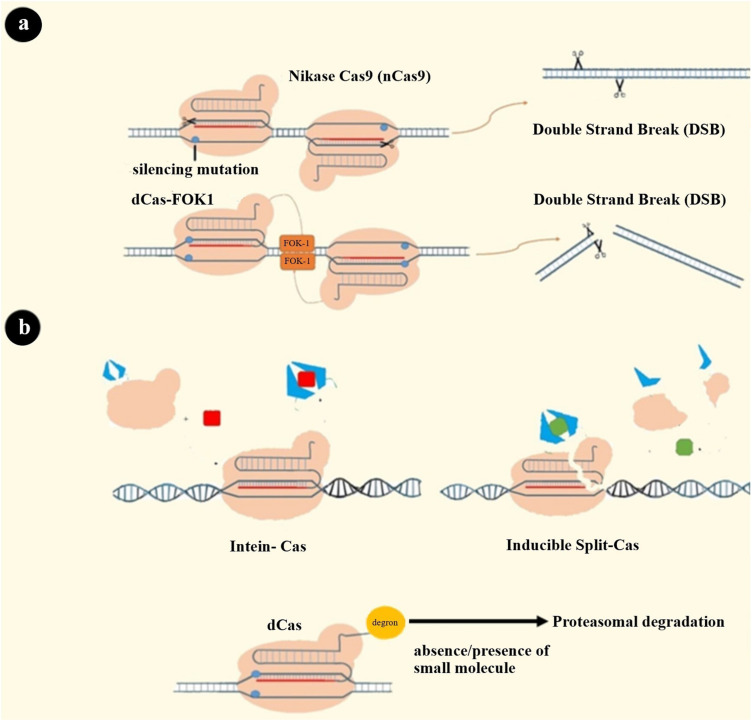
Some strategies to solve CRISPR system limitations. **(a)** Systems that need dimerization to cleave both strands: 1. dCas-FOK1 2. nCas9. **(b)** Cas9 variants’ activity can be switched on and off by cell-permeable molecules: (1) Intein-Cas9 is activated by excision of the intein bound to a specific position in Cas9. A cell-permeable small molecule induces this excision (189, 211). (2) Split-Cas9 is a Cas9 molecule that has been split into two fragments, and these two can be dimerized via drug-binding dimerization domains and a cell-permeable drug (212). (3) Degron-Cas9 is formed by binding a destabilizing domain (degron) to Cas9 protein. Previous studies have introduced a different type of degron that can bind to the protein of interest and decrease the stability of that protein in the presence or absence of specific small molecules. Degron domains can also be fused to the RBP-effector complex to regulate its stability and activity of the CRISPR system as a result (190, 213–216).

The choice of nuclease is another famous gate through which enhanced specificity can be achieved. Cpf1 and fnCas9 have been reported to have higher specificity than SpCas9 (174, 175). Changing amino acid residues for reducing OTEs has led to engineering high fidelity Cas9 proteins such as SpCas9-HF1, eSpCas9, and HypaCas9. The mechanism of action of these altered Cas9 proteins might be a change in nuclease domain activation pattern (HNH domain) or change in the strength of binding to target DNA (176–178). In an attempt to construct a SpCas9 that can recognize a broad range of PAM sequences, several Cas proteins with reduced OTEs were created (179, 180). Another form of nucleases was developed by fusing dCas to the FOK1 cleavage domain that generates a chimeric protein that needs dimerization for DNA cleavage. Since two closely located sequences’ recognition is needed through two distinct sgRNAs, this approach theoretically increases specificity (181). There is a similar strategy using nickase Cas9 that has a mutation in one of two nuclease domains of Cas9 protein. As a result, each nCas9, which is guided with a separate sgRNA, cleaves only one strand of DNA (Figure 4A) (182).

Limiting exposure time to CRISPR nuclease is the third primary strategy for reducing OTEs, which may have a concomitant reduction in on-target efficacy (171). Integrase-deficient lentiviral vector, ribonucleoprotein (RNP) complexes, and Cas9 mRNA are all delivery systems generated for shortening Cas9 exposure (183–185). Despite the many advantages of using the RNP delivery system, immune adverse events must be noted (186). Other strategies to limit this exposure time are: (i) using doxycycline-inducible promoter controlling Cas9 expression in order to have a regulated expression; (ii) creating Cas9-intein, Cas9-degron, and split Cas to regulate Cas9 activity through cell-permeable compounds (Figure 4B); (iii) designing a self-restriction construct consisting of a Cas9 that targets the system’s gRNA; and (iv) using a Cas9 natural inhibitor (e.g., AcrIIA4) (187–193).

## Discussion and Future Prospects

CRISPR/Cas9 has driven a paradigm shift in cancer treatment approaches since the day it was introduced. As mentioned above, despite sublime influences given by the CRISPR/Cas9 system in gene therapy, there are still many challenges that have to be considered.

The alternative Cas13 has been developed regarding the acknowledged challenges of Cas9-based CRISPR systems, including the risk of off-target toxicity, affecting wild type transcripts, and high molecular weight. Cas13 binds and cleaves single-stranded RNAs rather than DNA; therefore, it reduces the risk of off-target toxicity and wild type transcripts alterations. Moreover, the Cas13d subtype is known to maintain one of the lowest molecular weights among Cas enzymes. Thus, it can be introduced in target cells more easily through viral vectors. In conclusion, these RNA-targeting Cas enzymes would result in a considerable apprehension over transcriptome and RNA regulators functions in cancer cells (194). Besides, many types of other CRISPR related nucleases have been introduced, including Cpf1 and fnCas9 and nucleases that were made by changing amino acid residues in SpCas9 (SpCas9-HF1, eSpCas9, and HypaCas9) (174, 175). Application of these nucleases in cancer treatment and their probable advantages over more-studied SpCas9 should instead be evaluated in future studies.

Recent innovations in CRISPR-based systems have led to the emergence of new promising applications in cancer therapies. For instance, CDetection, as a CRISPR/Cas12b-based DNA detection system, has been developed to ease precise and sensitive DNA detection. Genetic variations, including Single Nucleotide Polymorphisms (SNPs), are also considered as cancer (especially sporadic cancers) etiologies (195, 196). Since the association of various SNPs with different cancers is well-established, precise DNA detection and its application in SNP genotyping would be favorable in the early clinical diagnosis of primary cancers. This precise DNA detection system is estimated to be able to detect over 20,000 known human disease-associated point mutations (197).

In summary, CRISPR and the above-mentioned advantages over other genome editing techniques could pave the road for cancer treatment in the future. This objective could be accomplished via using CRISPR as a potent instrument for gene therapy and identification of prognostic and predictive biomarkers, novel signaling pathways and targets, and new drugs in cancer treatment. CRISPR could also be used in identifying novel immune system-tumor interplays and augmenting cellular immunotherapies; however, related limitations and cautions should be noted before any interventions.

## Author Contributions

MA-K, MG, JKh, MB-S, and MJ performed the literature search and data analysis, drafted, and revised the work. MS and JKi critically revised the work. All authors contributed to the article and approved the submitted version.

## Conflict of Interest

The authors declare that the research was conducted in the absence of any commercial or financial relationships that could be construed as a potential conflict of interest.
